# Postgraduate career intentions of medical students and recent graduates in Malawi: a qualitative interview study

**DOI:** 10.1186/1472-6920-12-87

**Published:** 2012-09-14

**Authors:** Nicola Bailey, Kate L Mandeville, Tim Rhodes, Mwapatsa Mipando, Adamson S Muula

**Affiliations:** 1 Faculty of Health and Social Care Sciences, St Georges University of London and Kingston University, Terrace, Cranmer SW17 ORE, London, UK; 2 Faculty of Public Health and Policy, London School of Hygiene and Tropical Medicine, 15-17 Tavistock Place, London, WC1H 9SH, UK; 3 Department of Physiology, University of Malawi College of Medicine, Blantyre 3, Chichiri, Malawi; 4 Department of Community Health, University of Malawi College of Medicine, Blantyre 3, Chichiri, Malawi

**Keywords:** Doctors, Medical students, Postgraduate education, Specialisation, Malawi, Rural health, Brain drain, Emigration

## Abstract

**Background:**

In 2004, the Malawian Ministry of Health declared a human resource crisis and launched a six year Emergency Human Resources Programme. This included salary supplements for key health workers and a tripling of doctors in training. By 2010, the number of medical graduates had doubled and significantly more doctors were working in rural district hospitals. Yet there has been little research into the views of this next generation of doctors in Malawi, who are crucial to the continuing success of the programme. The aim of this study was to explore the factors influencing the career plans of medical students and recent graduates with regard to four policy-relevant aspects: emigration outside Malawi; working at district level; private sector employment and postgraduate specialisation.

**Methods:**

Twelve semi-structured interviews were conducted with fourth year medical students and first year graduates, recruited through purposive and snowball sampling. Key informant interviews were also carried out with medical school faculty. Recordings were transcribed and analysed using a framework approach.

**Results:**

Opportunities for postgraduate training emerged as the most important factor in participants’ career choices, with specialisation seen as vital to career progression. All participants intended to work in Malawi in the long term, after a period of time outside the country. For nearly all participants, this was in the pursuit of postgraduate study rather than higher salaries. In general, medical students and young doctors were enthusiastic about working at district level, although this is curtailed by their desire for specialist training and frustration with resource shortages. There is currently little intention to move into the private sector.

**Conclusions:**

Future resourcing of postgraduate training opportunities is crucial to preventing emigration as graduate numbers increase. The lesser importance put on salary by younger doctors may be an indicator of the success of salary supplements. In order to retain doctors at district levels for longer, consideration should be given to the introduction of general practice/family medicine as a specialty. Returning specialists should be encouraged to engage with younger colleagues as role models and mentors.

## Background

In 2004, Malawi had the lowest doctor staffing levels in southern Africa, with 1.1 doctor per 100,000 people [1]. There was huge disparity in distribution of staff between urban and rural areas. Whilst over 80% of Malawi’s population resides in rural areas, half of Malawi’s doctors at that time worked in central hospitals and 16 out of 23 district hospitals did not have a single doctor [2,3]. There were few Malawian-born specialists, with most specialist posts running at 80-90% vacancy and many positions filled by expatriate doctors [3-6]. Palmer [3] attributed this situation to five causes: 1) Inability of the state to train and employ enough health care workers (HCWs); 2) HIV/AIDS related attrition amongst health workers; 3) Movement into the private sector; 4) Difficult working conditions, poor resources and limited career opportunities; 5) International migration [2,3]. The Malawian Ministry of Health (MoH) declared a human resource crisis in 2004 and launched the Six-Year Emergency Human Resources Programme (EHRP) in 2005 [7], which attracted major donor funding and aimed to equal Tanzanian staffing levels of 2.3 doctors per 100,000 population (considered an attainable target) by 2010 [6,8]. The programme included a 52% taxed salary top-up for health workers and a 50% expansion in domestic training capacity [6].

Now at the end of the EHRP, Malawi’s doctor to population ratio has reached 2.03 per 100,000 [6]. Although still far from the World Health Organization recommendation of 1 doctor per 5000 people [9], Malawi has increased its doctor numbers by 516% in the last five years [6]. Through donor funding and government subsidised fees, Malawi’s College of Medicine (COM), has increased its number of graduating doctors from 18 in 2004, to 51 in 2011, and is enrolling more students every year. The MoH has created new posts for newly registered doctors in district hospitals and there is now at least one medical doctor in almost all district hospitals [6,10], although an intended rural hardship allowance has not yet been implemented [6]. A recent evaluation [6] suggests health worker migration has slowed since implementation of the EHRP: 16 nurses emigrated in 2009 compared with 108 in 2003. Little data is available on how doctor emigration has changed since the EHRP’s implementation [6]. In order to provide postgraduate education in Malawi rather than sending doctors to the UK to train, COM launched a Masters of Medicine (MMed) clinical specialist training programme in certain specialties [4]. For the majority, training is undertaken in both Malawi and South Africa, where visa restrictions prevent residence after completion of training [4].

Within this dramatically changed employment landscape, there has been little research into the views of the next generation of doctors in Malawi. The success of the EHRP rests on convincing these young doctors to remain in Malawi, yet little is known about what factors influence their career intentions and whether these have been affected by the EHRP’s impact. The aim of this study was to explore the factors influencing the postgraduate plans of medical students and recent graduates with regard to four policy-relevant aspects: 1) emigration outside Malawi 2) working at the district level 3) private sector employment and 4) postgraduate specialisation.

## Methods

### Design

This was a qualitative study using semi-structured interviews, as appropriate for an exploratory study with some pre-existing topics for discussion [11].

### Setting

**Figure 1:**
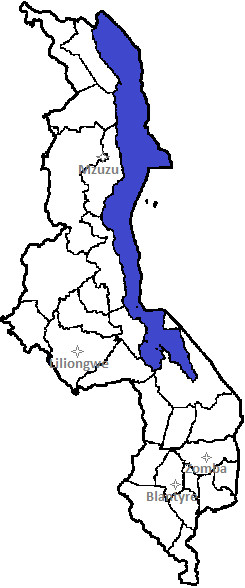
Map of Malawi showing main urban centres.

Malawi had a gross national income of USD 810 per capita in 2008 [1] and 81.2% of the population live in rural areas [1]. The government spends around 9.3% of total government expenditure on health and provides 60% of public health services, with the Christian Health Association of Malawi (CHAM) and private sectors providing the remaining 40% [6]. There are four central hospitals, 28 district hospitals, 23 mission hospitals run by CHAM on a not-for-profit basis, two psychiatric hospitals (one government and one CHAM) plus smaller health centres, posts and dispensaries in rural areas [3]. Junior doctors are posted down to the level of district hospitals, and specialists in the public sector are concentrated in the two teaching hospitals, Queen Elizabeth Central Hospital (QECH) in Blantyre and Kamuzu Central Hospital (KCH) in Lilongwe [10] (Figure 1). Mzuzu and Zomba Central Hospitals and selected mission hospitals may have between 1 and 5 specialists at any one time, although these are often international volunteers. There are several private hospitals based in the main cities and increasing numbers of private clinics, however overall Malawi’s private health sector is small compared to neighbouring countries.

Until 1991, Malawian medical doctors were sent abroad for training. The establishment of COM in Blantyre in 1991 allowed medical students to be trained in country for the first time [12]. After qualification, graduate doctors complete an 18-month internship in either QECH or KCH. Their salary is around USD 7000 per year, excluding housing. After this, they are allocated by the government to one of the 28 district hospitals. Whilst originally this was as a district health officer [4], the MoH has created the new post of district medical officer due to the increasing number of graduates. This post is junior to the district health officer and is mainly clinical in nature, while the former is largely administrative. There is a requirement to complete a minimum of two years at district level before being eligible to apply for government scholarships for specialty training.

Postgraduate training in the major specialties is provided through the Masters of Medicine programme at COM, however most of these require a period of training outside Malawi as part of the programme. Training in subspecialties, for example in oncology, is undertaken entirely outside Malawi. Family medicine (general practice) is not currently a recognised specialty in Malawi. A limited number of government training scholarships have been made available over the past few years, usually funded by donor agencies. Reduced funding means that the future number of scholarships is not certain.

### Study participants

The study participants were QECH interns and COM fourth year medical students. Fourth year students rather than final year students were selected as it was hypothesised that they would have considerable clinical exposure by that stage of their medical education that would allow them to have constructed views on their career plans, yet still have different perspectives to interns. By the fourth year, medical students have completed their initial clinical attachments and are now rotating around specialties. A mixture of purposive and snowball sampling was used for this study. Two interns and four fourth year medical students were purposively selected on their background characteristics. They were then each asked to nominate one peer to be interviewed. As this was an exploratory piece of work, intended to generate hypotheses for future studies, it was felt that interviewing twelve participants would be adequate. If new themes were still emerging after twelve interviews, the researchers planned to continue up to sixteen interviewees. In addition, in order to triangulate the results, seven COM faculty members were also interviewed as key informants, including non-clinical lecturers and clinical staff who also practise at QECH.

### Conduct of interviews

All interviews were undertaken in English and lasted twenty-five to forty-five minutes. Participants filled in a demographic information form prior to the interview, with clarifications made during the interview. The main topic guide was informed by a literature review and initial discussions between the authors. It included questions on students’ career aspirations and intentions, initially focusing on emigration, rural/urban working and private/public sector. A separate theme of postgraduate specialisation was added after initial interviews, as it was clear this topic deserved greater attention. A separate topic guide was developed for key informant interviews, with questions centred round observed trends in student/graduate career choices and perceived explanatory factors.

### Analysis

All interviews were tape-recorded and transcribed. Data analysis was conducted using a framework analysis approach. Framework analysis aims to produce policy-orientated findings and involves summarising data within a thematic framework [11,13]. It facilitates a comprehensive and systematic analysis. Here, transcripts were initially coded according to pre-identified themes identified in the policy and literature review. As new themes emerged in the transcripts, new codes and sub-codes were created. Emerging codes and hypotheses were discussed amongst the research team until consensus was reached.

### Ethical approval

The study was granted ethical approval by the London School of Hygiene and Tropical Medicine in the UK and the College of Medicine Research and Ethics Committee (COMREC) in Malawi. Written informed consent was obtained from all participants.

## Results

Participant characteristics and their career intentions are summarised in Table 1. Specific specialty choice has been omitted where we felt it could identify participants.

**Table 1:** Summary of participants’ career intentions

**Participant**	**Year of study**	**Gender**	**Emigration intentions**	**District/central location**	**Private/public sector work**	**Postgraduate specialty**
1	4	M	Study in Africa, work in Malawi	District then specialise so work central	Possibly in the future	Surgery
2	4	M	Study in Africa, work in Malawi	Prefer central hospital	Not mentioned	Surgery or Anaesthesia
3	4	M	Study in Africa, work in Malawi	District then specialise so work central	Not mentioned	Obstetrics and gynaecology
4	4	M	Study in Africa, work in Malawi	Prefer district but wants to specialise	Some discussion of private practice in surgery	Public health
5	4	M	Study in Africa, work in Malawi	District then specialise so work central	May set up own clinic after specialising	Medicine subspecialty
6	4	M	Study in Africa, work in Malawi	Prefer district but wants to specialise	Not mentioned	Surgery
7	4	M	Study in Africa, work in Malawi	Specialise then work in district	Not mentioned	Medicine subspecialty
8	4	F	Study in Africa, work in Malawi	Prefer district but wants to specialise	Mentioned it is easier for doctors in capital to find private work	Internal medicine
9	Intern	M	Study in Africa, work in Malawi	Prefer central hospital	No – wants to work for Government	Obstetrics and gynaecology
10	Intern	M	Study in Africa, work in Malawi	Prefer district but wants to specialise	Not mentioned	Surgery
11	Intern	F	Study in Africa, work in Malawi	Prefer central hospital	Not mentioned	Paediatrics
12	Intern	M	Work abroad then in Malawi	Flexible but wants to specialise	Will stay in government for postgraduate training opportunities	Medicine subspecialty or Public Health

### Intentions for Future Employment

All participants said they would like to study/work abroad for some time and then return to work in Malawi. Most thought they would work for the government for the foreseeable future. Although three students had considered practicing privately in the future, all interns wanted to work for the government. No participant expressed an interest in working for NGOs or any private sector organisations.

All participants wanted to undertake postgraduate training to specialise in the future. Three of the four graduates would prefer to start postgraduate training as soon as possible, rather than spending time working in government district hospitals first. Conversely, seven of the eight students said they would rather get experience working in district hospitals before specialising.

### Overall perception of current doctor emigration

Amongst students, graduates and staff, there was a feeling that emigration is less of a problem in Malawi now than it has been previously, and although some doctors will still leave, more are returning from postgraduate studies abroad: “Nowadays people are getting a different mentality and they want to work in their home country” (student). Some participants thought the same numbers of doctors were emigrating, but “because we have improved on the numbers [of doctors graduating] we think that it’s not a lot of doctors that are going out” (graduate). A number of students also commented on the fact that there are supposed to be more doctors in Malawi now, but “you still find that in some district hospitals you don’t have enough doctors. And you wonder, where did all those people go to?” (student). Participants thought most of their peers would leave for postgraduate training and then return to work in Malawi, and graduates noted of doctors who had gone abroad to specialise, “most of them, I see them come back” (graduate). Amongst all respondents, the main reason cited for doctors leaving Malawi was the possibility of higher salaries abroad, which could better support their families or pay them through specialist training: “After I graduate I will want to earn a good living so I can help my family out…here in Malawi our doctors earn a lot of money but you can’t compare it to the way you can earn money outside Malawi” (student).

Other reasons given for emigration include feeling disillusioned at being unable to use specialist skills due to lack of resources (“they might want to come back but maybe the specialty they did is not available here, and it’s difficult for them to contribute, so they may just opt to stay” – graduate); perception of doctors still not being sufficiently rewarded (“It’s improving but not the way it’s supposed to be like” – student); becoming accustomed to better conditions abroad and therefore feeling unsatisfied in Malawi, and being stuck working in a remote district hospital. A number of explanations were given for why fewer doctors are now emigrating. The first is remuneration – doctors’ salaries in Malawi have increased, and the government are offering newly graduated doctors “positions of authority and also I’d say finances as well” (student) in district hospitals. Other explanations were as follows: perception of stricter immigration rules for health professionals in the UK; sending students to South Africa for postgraduate training where their visa will not allow them to work afterwards; as there are few specialists in Malawi, “if we say we will specialise it means we have a good chance of employment” (student); doctors are now trained within Malawi and therefore have less links abroad; and “the magnet effect… when people were going away everybody wanted to go away, but now that people have seen people coming back and living quite comfortably, then they say, ok, if they have done it, then I can possibly do it too” (lecturer, COM).

### Factors affecting emigration choices

All participating students and graduates intended to work in Malawi eventually, however all also intended to spend time studying (eleven participants) or working (one participant) abroad first. Reasons participants gave for wanting to study or work abroad were centred around experience and exposure to a different health system and more advanced equipment, and in order to bring back valuable skills to Malawi: “I would maybe work for some years in a certain country just to see how things are, like maybe what the health system is like, but eventually I will come back to my country” (graduate). It was also recognised that training in certain specialties cannot be provided in Malawi and necessitated time overseas: “I would like to work in Malawi. I’ve never thought really about working in another country. I would love to work in Malawi but if I want to specialise in [medicine subspecialty] or [medicine subspecialty] then you always have to go abroad just to specialise and then you come back here to work” (student).

Reasons participants gave for wanting to return to work in Malawi were a desire to increase the number of doctors, patriotism and a love for their country, and recognition of Malawi’s significant health needs leading to an aspiration to “help my people” (student).

### Factors affecting urban/district working

The most common reason given by participants for working in an urban area is a desire to specialise, and therefore an assumption that afterwards “you have to work at a very big hospital” (student). Even those who expressed a desire to work at district level (i.e. in a district hospital) thought it unlikely they would be able to after they had specialised: “It depends on the resources, because I can say I can work in the district hospital but are there theatres there? I may not be efficient to the community because I’m just there but I don’t have the resources to work with” (student).

Poor living conditions and services, and frustration with lack of resources at district level were reasons to stay urban, as well as learning opportunities and exposure to complex cases at central hospitals: “At [QECH] there are a lot of consultants, so you get to learn more…as well as exposure to some of the conditions which you will not have in any other hospital” (student).

Almost all the students and two of the graduates expressed an interest in working at district level for some time. The main reasons were to “associate with people in the districts”(student), to get experience of cases which don’t usually reach central hospital, and in order to be eligible for government postgraduate funding (“people are assured that after working for the government for a while, they’ll get their scholarships” - graduate). Two students expressed a desire to improve health in the districts their families came from: “You have this passion you know; this is where I come from, and you want to improve things with all your might” (student). Others include: increasing the number of doctors in the districts and improving rural health; personal development (“you get mature because you’re managing the whole hospital by yourself” - graduate); exposure to administration and hospital management; and the fact that district posts are now relatively well paid and “more like a top position in government” (graduate). Most participants agreed doctors should spend some time working at district level once they finished medical school, because “the government is spending a lot to produce doctors…we should pay back by helping those people” (student). However, two participants thought “everybody must have the freedom to do what they want” (student) after their internship, and thought it was the government’s responsibility to encourage doctors to work in those areas: “The government funded them that’s true. But the working conditions shouldn’t be that bad so that somebody shouldn’t enjoy the rest of their career” (student).

Challenges associated with working at district level were the “huge workload” and “the challenge that everyone else will be looking up to you and say, we need a decision from you” (graduate). Other difficulties included the focus on administration duties over clinical practice; the maldistribution of staff and resources leading to posts in some districts being more challenging than others (“there are other districts which are very far and there aren’t good resources and the houses are not proper houses” – student); the fact that “In most districts there are not good opportunities to work in a private hospital” (student); and poor housing and living conditions.

Reasons given by participants who expressed an interest in working at district level after specialising focused on improving rural health by increasing the number of doctors at district level. One participant mentioned being from a rural area as a key motivation for working at district level (“I’ve seen how people struggle here, and I’ve struggled once in my life and there has to be a change somehow” – student); another mentioned the challenging environment as a motivating factor. These are participants who want to undertake postgraduate study and therefore, although motivated, believe they will be unable to work at district level afterwards due to lack of equipment.

Most staff thought that new posts at district level were encouraging doctors to continue working in the districts, due to improved salaries and the opportunity to make extra money from *per diems* given for meetings attended as senior MoH staff. Even so, it was thought once they married they would also want to move to cities (“their children should go to good schools” - lecturer).

### Factors affecting public/private sector working

There was no difference between students and graduates in their perceptions of the private sector, but one graduate suggested “the problems we face as doctors…so many responsibilities, you want to support your family, and you’re not getting enough money”, led many to practise privately. Most participants thought they would work for the government, at least initially, due to better postgraduate training opportunities, and discussion arose about doctors working in the public and private sectors simultaneously, or starting in the public sector and moving to the private sector once specialised: “I will practise in public, so in the government. But maybe I will also be having my own clinic somewhere, for sure” (student). Participants were not interested in working for NGOs as it was considered not to be a “long term career opportunity” (graduate). Some participants thought CHAM posts were better paid than government positions, and mentioned postgraduate training and better funded hospitals in CHAM as attractions; others thought the new government district posts were better paid. There seemed to be a perception amongst participants of being better looked after when working for the government than in the private/NGO sector.

Amongst staff, opinion was divided as to whether private practice, NGOs and CHAM were draining doctors from the public sector or not – some perceived it to be a “big drain”, others stressed that the private sector in Malawi was very small and therefore not employing many doctors, and that training opportunities were better with the government. A senior lecturer at COM noted “the Malawi thinking is that if you end up working in private sector but still in Malawi, you’re not really a loss to the nation because you’re still serving Malawians.” It was acknowledged that due to low pay, many senior staff at COM and QECH engage in dual practice, and that switching between public and private practice was frequent: “it’s not rigid, people change” (lecturer).

### Factors affecting postgraduate specialisation

All participants wanted to specialise in the future. The main reason given for wanting to specialise was ‘upgrading’; almost all participants wanted to further their education and not stay at a generalist level. The medical degree was seen as a ‘first degree’ which needed ‘upgrading’ for career progression. “It doesn’t make sense just to stay on the same level. I think it’s not even recommended for doctors just to stay on the same level. You need to do some upgrading” (student).

This aspiration to specialise often curtailed time working at district level, “you might want to work in the district hospitals but then at a certain point in time you would want to specialise” (student), and for four participants took priority over a preference for district work (Table 1).

Other reasons for specialising were to provide better care to patients (“You need to specialise, get yourself attached to one field. In so doing you can provide better treatment to the patients that you see” – graduate) and improved work satisfaction: (“If you are someone who has specialised, I think Malawi is a better environment to work in. Why? We tend to see lots of patients here so you would find your work interesting. You wouldn’t get bored. There are always customers” – graduate). Two students were motivated by being one of very few specialists in their field, and one explained they would like to inspire others to go into that field. “I think it’s an exciting prospect to be among the very few. Maybe if I will become a neurologist, I would inspire other people who would maybe also want to be a neurologist” – student. Interestingly, none of the graduates mentioned a lack of specialists in their chosen field as a motivation.

A major concern for all participants, but particularly graduates, was procuring funding for postgraduate study. “There is such a long waiting list. It depends how much patience you have and resilience. Some people would just stop [searching for a scholarship]” (student). All graduates were anxious about the limited number of government scholarships available. “There a lot of people right now in the department who are registrars who wants to go to school, but because the government doesn’t have enough money, then they are in like a queue, they send only like two people, one or two at a time, so I think it’s quite difficult.” (student). Three graduates intended to look for private scholarships so they could specialise as soon as possible, rather than working at the district level for two years. “If I had other options of like getting a better job, securing money so that I can sponsor myself, I would have grabbed such an opportunity” (graduate). Reasons included a desire to complete postgraduate training whilst they have few other commitments (“Right now I’m not married, so before I get too involved with that I want to do postgraduate training” - graduate) and a sentiment that further training was easier to undertake whilst still young and “the brain is fresh” (graduate). One graduate stated that he was considering emigrating to earn enough money to fund himself through postgraduate study, if he was unable to secure a scholarship: “I would love to stay in Malawi. But am I going to secure a scholarship? That’s the main worry”.

All participants had a clear idea of their intended specialty. The choice of specialty, for many participants, was based upon role models amongst doctors practising in Malawi: “Our consultants are like our role models - that would be one of the things which encourages people into what specialty they want to go” (student). This visibility is reflected in the range of intended specialties, with the major specialties of internal medicine, surgery and obstetrics and gynaecology well represented but few subspecialties selected (Table 1). Participants also mentioned the burden of disease in Malawi as a factor in their choice: “I just have this idea that it’s good to put effort where it’s needed most. If there’s an area which needs a lot of effort in Malawi, it’s obs and gynae.” (student). However, both students and graduates were aware that the government had moved towards a policy of training more subspecialties in underrepresented fields: “So far, most of the doctors that had trained previously, they were doing things like general medicine, surgery, paediatrics, obs and gynae, but now they are more oriented on other specialties which haven’t been covered so far” (graduate). Despite this policy shift, only one participant recognised that s/he may not be able to train in their first choice specialty: “For example, the government has said there is an oncology scholarship, you never planned on doing oncology, but since you can’t secure a scholarship in an area of your interest you are forced to pick something which is different all together.” (graduate). Two staff members felt that it was useless to train specialists in certain fields if the required equipment was unavailable in country for them to practise afterwards, e.g. radiotherapy for oncologists. One member of staff commented on the need for recognised postgraduate training in family medicine/general practice, so doctors felt they were furthering their education but could then practise general medicine. All staff agreed that career advice and guidance was lacking for students and graduates at present, but that this situation should improve as more specialists return to Malawi from training abroad.

## Discussion

Postgraduate training opportunities emerged in this study as the most important factor in Malawian medical students’ and graduates’ career choices, alongside the importance of adequate resources and equipment for doctors to practise without becoming de-motivated and leaving the public sector, and the value of role models. There is a general perception that fewer doctors are emigrating than before, with higher remuneration and the magnet effect of more doctors enhancing retention. This is the first study, to the best of our knowledge, to explore the views of Malawian doctors since the end of the EHRP.

The desire to specialise affected participants’ career decisions in a number of ways. Firstly, all participants intended to work for the government during their early career due to greater availability of postgraduate training scholarships in the public sector. After internship, most participants intended to spend time working in district hospitals to be eligible for these postgraduate scholarships. Even if they preferred working at district level, they would cut this short in order to start postgraduate training. If becoming a specialist is considered as the natural progression in a medical career it is perhaps unsurprising that medical graduates would aspire to work at district level (and leave ahead of time) in order to access postgraduate training opportunities afterwards. Participants generally acknowledged that once they had specialised they would be unable to work at district level due to a lack of specialist posts, resources and equipment, and therefore virtually all expected to be based in an urban centre after postgraduate study. Other studies have identified specialising as a potential barrier to choosing rural practice [14,15]. Developing general practice/family medicine as a specialty in Malawi could provide an opportunity for career progression for those doctors interested in working at district level in the long term, although there is a risk that the prestige afforded to specialists in family medicine might be lower than that afforded to other specialties as seen in other countries. Whilst rotations in family medicine have been introduced at undergraduate level, there is no postgraduate training programme at present and little recognition of the value of generalist doctors.

Postgraduate study opportunities also affect participants’ intentions to emigrate – nearly all participants intended to study abroad for some time in order to gain more comprehensive specialist skills in higher-resourced environments. Several surveys of doctors living outside their country of training confirm that postgraduate training is a major reason for emigration [16-19]. Although all participants stated their intention to return to Malawi eventually, there is a risk that some will stay abroad after postgraduate study, particularly if they perceive it will be difficult to practise their specialty in Malawi [6]. There is an increasing discrepancy between the number of postgraduate scholarships available and the number of medical graduates in Malawi. Without a career pathway of equal status for those doctors who are unable to obtain scholarships, new doctors may emigrate in search of other opportunities for postgraduate training and specialist status.

Whilst the pursuit of higher salaries was recognised as an important factor in historical emigration, the higher remuneration of doctors under EHRP is perceived to have aided the retention of new doctors. Indeed, the lesser emphasis placed on salary in participants’ career plans could be interpreted as an indication that the EHRP salary supplements have satisfied this aspect of job satisfaction. There was little intention by participants to move into the private sector in the future, although some participants mentioned setting up private clinics after specialising. In general, students and graduates felt that the postgraduate training prospects offered in the government sector were an important factor in their early career choices. It must be acknowledged that postgraduate training will translate into monetary gains for junior doctors in the long-run, as obtaining specialist status leads to an increase in government salary and opens up opportunities for private practice alongside government service.

An important factor affecting participants’ intentions to work in Malawi, and to work at district level, is a desire to improve the health of the population. The importance of serving one’s “own people” has been discovered in previous studies [15,20-22]. It also influences some participants’ choice of specialty (e.g. one student wanting to specialise in obstetrics to improve maternal mortality in Malawi). Almost all participants mentioned the lack of doctors in their discourse about emigration or district working, and most participants expressed a sense of social responsibility or altruism to improve the health system in Malawi.

A final factor affecting participants’ career aspirations in this study is that of role models, and career advice. Many students were unsure how they would go about specialising in their chosen field, and potential career paths were unclear, even for graduates. Participants tended to be inspired by senior staff working in Malawi, and the idea repeatedly arose that students choose to specialise in fields they can see doctors already practicing in. The idea of ‘role models’ arises frequently in literature on student career choices, in particular referring to the lack of role models in general practice/family medicine, leading students to choose other fields [23,24]. Participants mentioned opportunities for learning from senior staff as a reason to work in central hospitals, and noted the importance of strong management to retain staff in district hospitals. The number of role models in Malawi should increase as more doctors return from postgraduate study abroad. Engaging these newly qualified specialists in career guidance and mentoring for medical students, alongside the expansion of the MMed programme at COM, should assist students with planning their careers. As suggested by Couper *et al.*[15] in the South African context, strong role models working at district level, along with the introduction of general practice/family medicine as a specialty, may encourage more students to work in rural areas in the future. Somers *et al.*[25] reflect on the importance of general practitioners in rural Australia advising students considering a career in rural practice, having already overcome many of the difficulties themselves. In the Malawian context, it is unlikely that doctors returning from postgraduate study abroad will work at district level, but the fact that they return may influence future graduates to stay in Malawi, as they will have greater perceived collective efficacy [26].

The factors influencing medical students compared to graduates differed to a certain extent – generally graduates were keen to secure a scholarship to specialise as soon as possible, whereas students seemed more enthusiastic about working at district level first. Graduates discussed factors like starting a family and continuing postgraduate study while their brains were “still fresh”, which perhaps indicates a greater awareness of time producing an urgency to finish training. This may also indicate a growing realisation that larger cohorts of medical students will be graduating soon after them, increasing the competition for postgraduate training opportunities. Changing aspirations of medical students throughout their careers has been explored in other studies, in particular that students become more realistic about training times as they get older, and tend to prioritise their family over more altruistic motivations [21,27].

### Limitations of the study

The findings of this study are very specific to the Malawian context, but they emphasise how important postgraduate training opportunities are to doctors, and how funding to undertake postgraduate study can be used effectively as an incentive to retain doctors in rural areas and countries of training.

Although the lead researcher was independent of the COM, participants may have given socially desirable responses due to her position as an outsider to both the medical fraternity and Malawian society. Their expressed views may also be different to both what they really think and to what they end up doing in reality, particularly as all participants had limited professional experience. For example, students who have clear intentions of returning to work in Malawi may feel differently once they have experienced health systems elsewhere, especially better resourced ones with more opportunities for specialists. There were noticeable differences between students’ and graduates’ perceptions of their options for the future, and therefore the data may have been more accurate if the study had focused on graduates only rather than students, as they are likely to be less idealistic [21] and are closer to making actual decisions about their future. Finally, the recruitment of study participants (purposive and snowballing) may have compromised the generalisability of the findings.

## Conclusions

This study has allowed an important insight into the possible effect of the EHRP on the career intentions of young doctors and doctors in training. Opportunities to pursue postgraduate training appear to now have more influence on future career plans than salary, and future resourcing of this area is crucial to preventing long term emigration. There seems to be little reason to worry about movement into the private sector at this stage. In general, medical students and young doctors are enthusiastic about working at district level, although this may be only for a limited time period and is likely to be fuelled (or curtailed) by the desire for specialist status. In order to retain doctors at district levels for longer, due consideration should be given to the introduction of general practice/family medicine as a recognised specialty in Malawi. Returning specialists should be encouraged to engage with younger colleagues as role models and mentors.

## Abbreviations

CHAM, Christian Health Association of Malawi; COM, College of Medicine, University of Malawi; EHRP, Emergency Human Resources Programme; KCH, Kamuzu Central Hospital; MMed, Masters of Medicine; MoH, Ministry of Health, Government of Malawi; NGO, Non-governmental organisation; QECH, Queen Elizabeth Central Hospital; UK, United Kingdom.

## Competing interests

The author(s) declare that they have no competing interests.

## Authors’ contributions

NB and KM conceived the study. TR and AM contributed to its design. NB carried out the primary data collection and initial analysis, with participant recruitment facilitated by AM and MM. KM, TR and AM helped with further analysis of the data. NB and KM drafted the manuscript. All authors read and approved the final manuscript.

## Authors’ information

MM is Faculty Dean and AM Deputy Faculty Dean at COM. KM runs a small non-governmental organisation, Medic to Medic, which supports students at COM. This research project, of which these results are a part, was submitted by NB as part of the requirements for a postgraduate Masters in Public Health at the London School of Hygiene and Tropical Medicine.

## Pre-publication history

The pre-publication history for this paper can be accessed here:

http://www.biomedcentral.com/1472-6920/12/87/prepub
